# Work-related physical and psychosocial risk factors cluster with obesity, smoking and physical inactivity

**DOI:** 10.1007/s00420-020-01627-1

**Published:** 2021-01-06

**Authors:** Mandy van den Berge, Allard J. van der Beek, Rukiye Türkeli, Marike van Kalken, Gerben Hulsegge

**Affiliations:** 1 Department of Public and Occupational Health, Amsterdam Public Health Research Institute, Amsterdam UMC, Vrije Universiteit Amsterdam, Van der Boechorststraat 7, NL-1081 BT Amsterdam, The Netherlands; 2 Bewegen Werkt, Welnalaan 5, 7523 NG Enschede, The Netherlands; 3 The Netherlands Organization for Applied Scientific Research, TNO, Schipholweg 77-89, 2316 ZL Leiden, The Netherlands

**Keywords:** Work demands, Occupational stress, Socioeconomic position, Lifestyle, Obesity

## Abstract

**Objective:**

This study investigated associations between the co-existence of multiple types of work-related psychosocial and physical risk factors, and (1) obesity; (2) smoking; and (3) leisure-time physical inactivity. It also aimed to identify sociodemographic characteristics related to clustering of work-related risk factors and lifestyle factors.

**Methods:**

Cross-sectional data on work-related risk factors (e.g., decision authority and repetitive movements) and lifestyle was measured using a standardized questionnaire among 52,563 Dutch workers in health care, services, manufacturing and public sector. Multiple-adjusted logistic regression models assessed associations between the co-existence of multiple types of psychosocial and physical risk factors and lifestyle factors. Additionally, logistic regression models related age, gender and educational level to clustering of risk factors and lifestyle factors.

**Results:**

The co-existence of multiple types of work-related psychosocial risk factors was associated with higher odds of smoking and being physically inactive. For example, workers exposed to three psychosocial risk factors had a 1.55 times higher odds of being physically inactive (95%CI: 1.42–1.70) compared to unexposed workers. A higher number of physical risk factors was also significantly associated with higher odds of smoking and obesity. The co-existence of multiple types of physical risk factors was not associated with higher odds of physical inactivity. Clustering of work-related risk factors and at least one unhealthy lifestyle factor occurred in particular among workers with low educational level.

**Conclusions:**

Results imply that interventions are needed that focus on workers with a low educational level and address work-related physical and psychosocial risk factors as well as lifestyle.

**Supplementary Information:**

The online version contains supplementary material available at 10.1007/s00420-020-01627-1.

## Introduction

The prevalence of obesity and unhealthy lifestyle behaviors among adults is high. In Europe, approximately 16% of the adult population is obese (Marques et al. 2017), 24% smokes (Reitsma et al. 2017), and 29% is physically inactive (Dumith et al. 2011; Sjöström et al. 2006). This high prevalence of obesity, smoking and physical inactivity are a major public health concern as they increase the risk of several chronic diseases, most notably type 2 diabetes and cardiovascular diseases (Danaei et al. 2009, 2014).

Both work-related psychosocial and physical risk factors are widely present at the workplace and might also be risk factors for the development of obesity and unhealthy lifestyle behaviors (Bambra et al. 2009; Häusser et al. 2010). According to the Demand-Control-Support model, a combination of work-related psychosocial risk factors, low decision latitude and low social support, cause occupational stress (Dawson et al. 2016; De Jonge and Kompier 1997). In turn, occupational stress has been found to be associated with obesity (Brunner et al. 2007; Jaaskelainen et al. 2015). For example, the prospective cohort study of Brunner et al. (2007) found a dose–response association between the exposure to occupational stress and the risk of obesity at follow-up (Brunner et al. 2007). Several other, mainly cross-sectional, studies have shown that work-related psychosocial risk factors, such as job strain and low decision latitude, are associated with smoking (Heikkilä et al. 2012; Radi et al. 2007), and physical inactivity during leisure time (Heikkilä et al. 2013; Kouvonen et al. 2013). For example, an individual-participant data meta-analysis based on cross-sectional and longitudinal data has shown that workers who reported high levels of job strain were 1.12 times more likely to be physically inactive during leisure time compared to workers without job strain (Heikkilä et al. 2013). According to several models regarding work-related musculoskeletal disorders, such as the conceptual model of the FINALE program (Holtermann et al. 2010), work-related physical risk factors increase the risk of musculoskeletal disorders partly via unhealthy lifestyle factors (van der Beek et al. 2017). Furthermore, previous cross-sectional studies found that workers with high physical workload were more inactive during leisure time than those with low physical workload (Mäkinen et al. 2010; Morassaei and Smith 2011; Schneider and Becker 2005).

Most of the studies investigated single work-related risk factors in relation to lifestyle factors. However, work-related risk factors often occur together and the co-existence of multiple types of work-related risk factors might even be more strongly related to obesity and unhealthy lifestyle behaviors compared to a single work-related risk factor. This has been confirmed in one cross-sectional study by Miranda et al., which showed that the co-existence of multiple types of risk factors was related to a higher risk of being overweight, a smoker, and physically inactive (Miranda et al. 2015). Thus, the co-existence of multiple types of work-related risk factors might cluster with unhealthy lifestyle factors among the same workers. From their study it is, however, still unclear whether this is the case for both physical and psychosocial risk factors, while the association with lifestyle factors may differ between these risk factors because of different underlying mechanisms (Nobrega et al. 2016). For example, work-related psychosocial risk factors are associated with smoking and alcohol use to relieve stress (Heikkilä et al. 2012), whereas work-related physical risk factors are associated with physical complaints and fatigue after work, which in turn increase the risk of physical inactivity during leisure time and obesity (Nobrega et al. 2016). Furthermore, the study population of the study of Miranda et al. (Miranda et al. 2015) was restricted to female nursing home workers. Thus, it remains unclear whether those results are to the same extent applicable to other occupational sectors where workers are exposed to other risk factors.

Prevention can either be based on a population strategy, in which all workers are targeted, or a high-risk strategy, in which the most vulnerable workers are targeted. For the latter strategy it is needed to identify subgroups of workers with the highest risk of health problems. It might, therefore, be helpful to identify subgroups of workers in whom multiple types of work-related risk factors and unhealthy lifestyle factors cluster. It might be that work-related risk factors and unhealthy lifestyle factors cluster among workers with a low socioeconomic position and less so among those with a high socioeconomic position. Previous work showed that work-related risk factors as well as lifestyle factors explained part of the socioeconomic health inequalities among workers (Dieker et al. 2019). Second, older workers with physically demanding work are at higher risk (e.g., for musculoskeletal disorders) and need a longer recovery time after work to avoid injuries and fatigue, compared to younger workers (Kenny et al. 2008). This can be explained by biological changes due to the ageing process, but also due to the fact that a higher number of years being exposed to physically demanding work tasks is associated with an increased risk of disorders (Buckwalter 1995; Törner et al. 1988). Due to a decline of overall cognitive resources and reserves by age, psychosocial risk factors may also be a higher risk for older workers than younger workers (Hansson et al. 2001). Third, the exposure and the experience of psychosocial and physical risk factors might be different among male and female workers (Cooper et al. 2011; Strazdins and Bammer 2004). Although previously mentioned studies indicate that relations between work-related risk factors and lifestyle are influenced by certain demographic factors, it is still unclear to what extent the co-existence of multiple types of work-related risk factors and lifestyle factors differ between these subgroups of workers.

Therefore, the present study aimed to investigate associations between the co-existence of multiple work-related psychosocial and physical risk factors, and (1) obesity; (2) smoking; and (3) physical inactivity during leisure time among Dutch workers. It is hypothesized that workers who were exposed to multiple types of work-related psychosocial and physical risk factors were more likely to be (1) obese; (2) a smoker; and (3) physically inactive during leisure time. The second aim was to determine the sociodemographic characteristics of the workers in whom multiple work-related risk factors and unhealthy lifestyle factors cluster. This study provides new insights into the extent that multiple types of work-related risk factors cluster with an unhealthy lifestyle. This gives direction to prevention strategies and the potential importance of an integral approach that focuses on specific groups of workers who are exposed to multiple types of risk factors and unhealthy lifestyle factors. Our study also gives indications regarding the specific groups on whom the integral prevention strategies might be focused.

## Methods

### Study population

Cross-sectional questionnaire data of the company ‘Bewegen Werkt’ (‘Moving Works’), which was gathered in enquiries among Dutch workers between 2009 and 2015 was used. These enquiries could have been part of an occupational health check, workers satisfaction screening or part of a vitality campaign in a company. Bewegen Werkt is an organization that promotes sustainable employability of workers and organizations with the focus on improving lifestyle. For the present study, we excluded 75 workers not in working age at the time of the measurements (younger than 18 years and 60 workers older than 65 years). In addition, 514 underweight workers (body mass index (BMI) < 18.5 kg/m^2^) were excluded, because underweight has been found to be associated with preexisting illnesses, such as cancer (Roh et al. 2014; WHO 2000). Another 436 (0.9%) participants were excluded due to missing data on sociodemographic or work-related characteristics, body weight, body height, and/or lifestyle behaviors. This resulted in a study population of 53,648 workers from the health care, services, manufacturing, and public sector. The present study was approved by the Medical Ethics Committee of VU University Medical Center.

### Outcomes

Body mass index (BMI) was calculated by dividing self-reported body weight in kilograms by the square of the self-reported body height in meters, and obesity was defined as a BMI of ≥ 30 kg/m^2^. Based on physical activity guidelines that recommend at least 30 min per day moderate-intensity physical activity on at least 5 days per week (WHO 2000), physical inactivity during leisure time was assessed using the following question with the response options yes and no: ‘’*Do you exercise approximately 30 min a day during your leisure time?’’.* Smoking was categorized as smokers or non-smokers based on the following question with the response options yes and no: ‘’*Do you currently smoke?’’.*

### Work-related psychosocial risk factors

The questions addressing self-reported work-related psychosocial and physical risk factors were derived from the Working Capacity Monitor (in Dutch: Monitor Duurzame Inzetbaarheid (MODI)) questionnaire (Alavinia et al. 2009; Hooftman et al. 2014). The questionnaire included three types of psychosocial risk factors. Decision authority was measured using the following five items: (1) influence on work tasks; (2) influence on planning; (3) ability to co-decide on work deadlines; (4) ability to decide on how to conduct work; and (5) ability to interrupt work tasks (Cronbach’s α = 0.82). Skill discretion was measured using the following three items: (1) required creativity in work; (2) required variety in work; and (3) required skills and abilities in work (Cronbach’s *α* = 0.65). Job demand was assessed using five items: (1) amount of work tasks; (2) time pressure at work; (3) working pace; (4) experienced problems with working pace; and (5) experienced problems with job demands (Cronbach’s *α* = 0.85). All items regarding psychosocial risk factors were answered on a 4-point Likert scale with the following anchors: ‘never’, ‘sometimes’, ‘often’, and ‘always’. Items were reversed appropriately, so that higher scores indicated a higher exposure to the psychosocial risk factors. For all three psychosocial risk factors, participants with a score in the upper quartile were defined as exposed to the psychosocial risk factor (Robroek et al. 2013). A standardized sum score ranging from 0 to 3 was calculated by summing all psychosocial risk factors.

### Work-related physical risk factors

The questionnaire addressed seven types of work-related physical risk factors. In this study, the items lifting or moving heavy loads of > 5 kg and > 25 kg were taken together as one physical risk factor. Participants were classified as exposed, if the item lifting or moving loads of > 5 kg was answered with ‘quite a lot’ or ‘a lot’, and/or if the item lifting or moving loads of > 25 kg was answered with ‘now and then’, ‘quite a lot’, and ‘a lot’. The items (1) awkward working postures; (2) applying force with arms or hands; (3) frequently bending and/or twisting the upper body; (4) frequently working in the same position; and (5) repetitive movements with arms and/or hands were all measured with a single item. All items regarding physical risk factors were answered on a 4-point Likert scale with the following anchors: ‘seldom or never’, ‘now and then’, ‘quite a lot’, and ‘a lot’. The participants were defined as exposed to the physical risk factors, if answered with ‘quite a lot’ or ‘a lot’. The standardized sum score ranging from 0 to 6 was calculated by summing all six physical risk factors.

### Sociodemographic and work-related characteristics

Age (continuous), gender (male/female), working hours per week (continuous), irregular working hours (yes/no) and educational level (intermediate secondary education or less/intermediate vocational or higher secondary education/higher vocational education or university) were self-reported.

### Data analysis

Logistic regression analyses were performed to assess associations between the number of work-related psychosocial and physical risk factors on the one hand, and lifestyle factors on the other. No exposure to psychosocial and physical risk factors was the reference category in all analyses. *P* values for trend were obtained by adding the categorical variables of psychosocial and physical risk factors linearly to the model. All analyses were adjusted for age, gender, working hours per week and irregular working hours, because of their association with work and lifestyle factors (Golden 2015; Winkler et al. 2018).

Potential sociodemographic characteristics associated with the co-existence of multiple types work-related risk factors and unhealthy lifestyle factors were analyzed for psychosocial and physical risk factors separately. To do so, we used logistic regression analysis relating age, gender and education in the same model to (1) the workers with 2–3 psychosocial risk factors and at least one unhealthy lifestyle factor that was linearly related to the number of psychosocial risk factors (*p* for trend was assessed) vs. all others; and (2) the workers with 4–6 physical risk factors and at least one unhealthy lifestyle factor that was linearly related to the number of physical risk factors vs. all others. To gain more insight in the underlying explanation of the results, effect modification was tested for the sociodemographic characteristics in the associations between work-related risk factors and lifestyle factors. In addition, the prevalence of the number of work-related risk factors and unhealthy lifestyle factors was calculated. *P* values were considered statistically significant if *p* < 0.05 and SPSS version 22.0 was used to conduct statistical analyses.

## Results

### Characteristics of study population

Participants were on average 43.7 years of age (SD: 10.8) (Table 1). Most participants worked in the health care sector (62%), were women (62%), and had an intermediate education (45%). 10% of the participants was obese, 20% was smoker, and 33% was physically inactive during leisure time. Low skill discretion was the most reported psychosocial work demand (42%), and frequently bending/twisting the upper body and frequently working in the same position were the most often reported work-related physical risk factors (both 40%).

**Table 1:** Characteristics of the participants

	Total (*N* = 52,563)
Sociodemographic characteristics	
Gender (female)	32,410 (61.7%)
Age (years)	43.7 ± 10.8
Educational level	
Low	7,863 (15.0%)
Intermediate	23,383 (44.5%)
High	21,317 (40.6%)
Overweight and lifestyle	
Obesity (BMI ≥ 30.0 kg/m^2^)	5,390 (10.3%)
Current smoker	10,431 (19.8%)
Physically inactive during leisure time	17,396 (33.1%)
Occupational sector	
Health care	32,381 (61.6%)
Services	3,822 (7.3%)
Manufacturing	10,674 (20.3%)
Public	5,686 (10.8%)
Working hours/week	29.7 ± 9.4
Irregular working hours	22,533 (42.9%)
Work-related psychosocial risk factors	
Low decision authority	15,452 (29.4%)
Low skill discretion	22,120 (42.1%)
High job demands	13,874 (26.4%)
Work-related physical risk factors	
Lifting/moving heavy loads	18,886 (35.9%)
Awkward working postures	8,930 (17.0%)
Applying force with arms/hands	11,646 (29.8%)
Frequently bending/twisting upper body	21,202 (40.3%)
Frequently working in the same position	21,166 (40.3%)
Repetitive movements with arms/hands	20,946 (39.8%)

Values represent means ± standard deviations, numbers and (percentages)

*N* number of participants; *M* mean; *SD* Standard Deviation; *BMI* Body Mass Index

### Work-related psychosocial risk factors

**Fig. 1:**
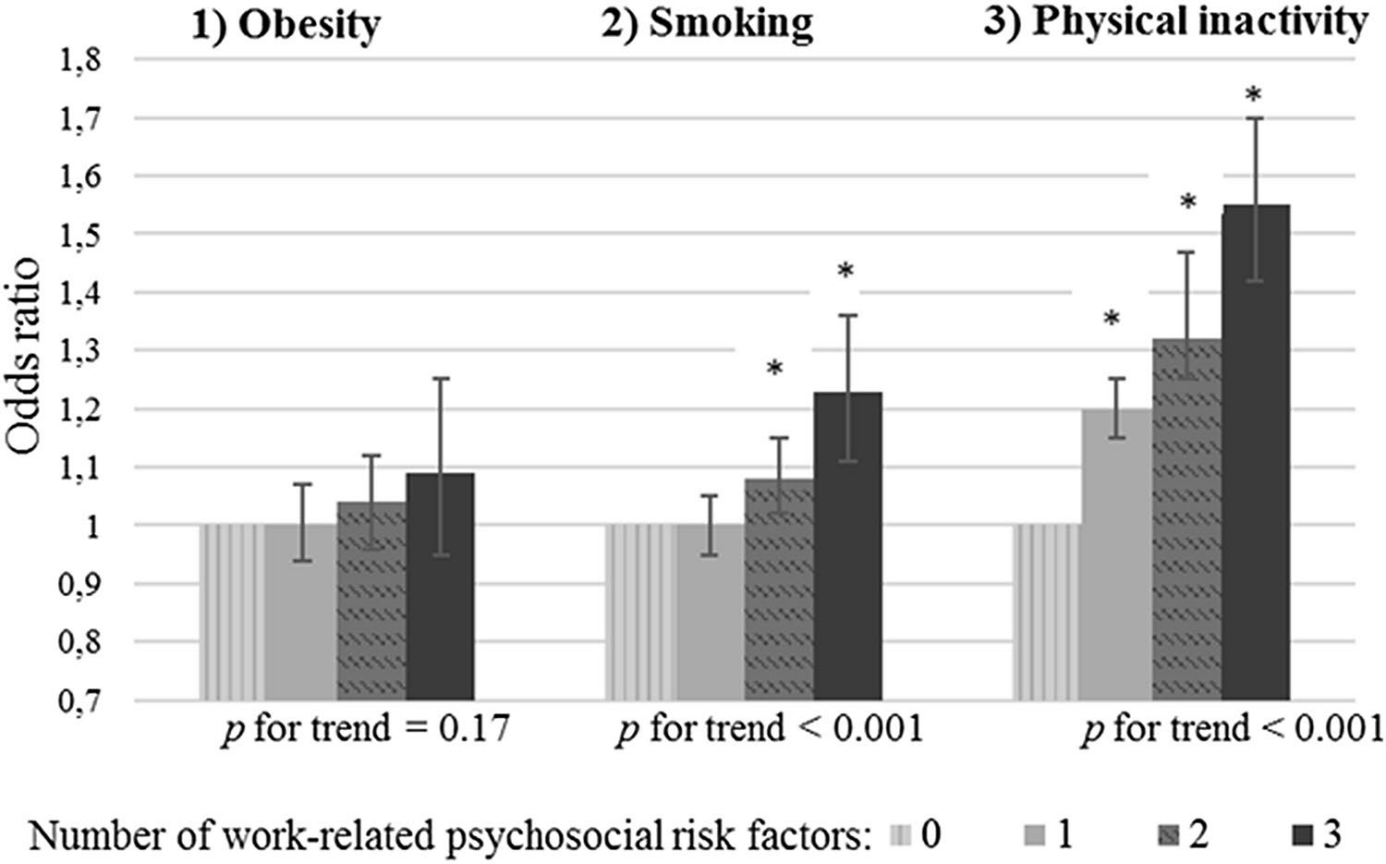
Associations between the number of work-related psychosocial risk factors and obesity (**a**), smoking (**b**), and physical inactivity during leisure time (**c**) in the total population (*N* = 52,563). Analyses were adjusted for gender, age, working hours per week and irregular working hours. Note: Sum of psychosocial risk factors: low decision authority, low skill discretion, and high job demands. *0* psychosocial risk factors = reference category *p < 0.05

Thirty-three percent of the participants reported that they were not exposed to work-related psychosocial risk factors, 41% to one, 21% to two, and 5% were exposed to all three types of work-related psychosocial risk factors. As shown in Fig. 1 and Supplemental Table 1, the more types of psychosocial risk factors that co-existed among workers, the higher the odds of being a smoker and being physically inactive (*p* for trend < 0.001). Workers exposed to three types of psychosocial risk factors had a 1.23 times higher odds of being a smoker (95%CI: 1.11–1.36) and a 1.55 times higher odds of being physically inactive during leisure time (95%CI: 1.42–1.70) compared to workers not exposed to psychosocial risk factors. No association was found for of the co-existence of multiple types of psychosocial risk factors and obesity (*p* for trend = 0.17).

### Work-related physical risk factors

**Fig. 2:**
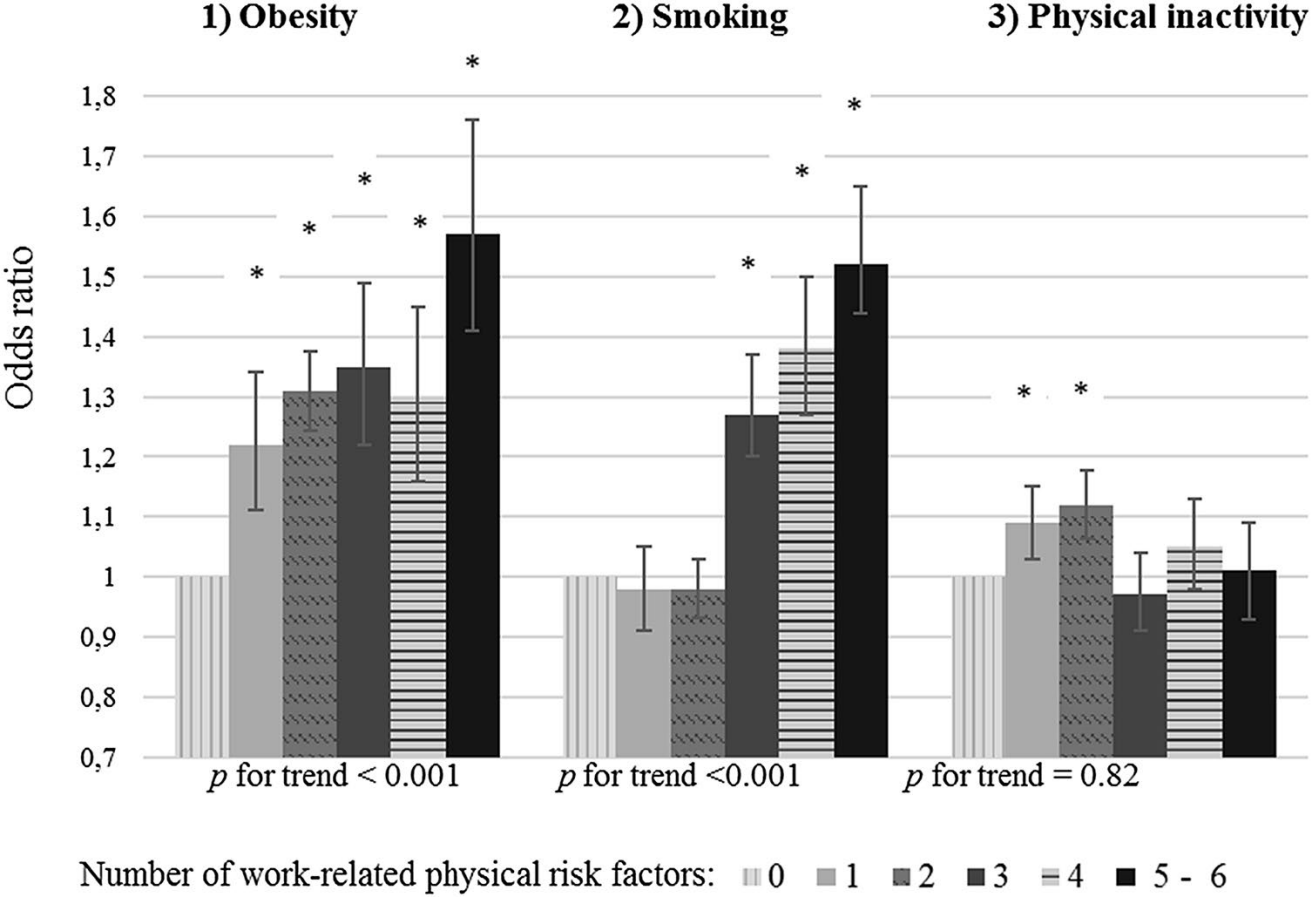
Associations between the number of work-related physical risk factors and obesity (**a**), smoking (**b**), and physical inactivity during leisure time (**c**) in the total population (*N* = 52, 563). Analyses were adjusted for gender, age, working hours per week and irregular working hours. Note: Sum of physical risk factors: lifting or moving heavy loads, awkward working postures, applying force with arms or hands, frequently bending and/or twisting the upper body, frequently working in the same position, and repetitive movements with arms and/or hands. *0* physical risk factors = reference category *p < 0.05

Twenty-one percent of the workers reported no work-related physical risk factors, 18% one, 27% two, 15% three, 10% four, and 9% reported five or six types of work-related physical risk factors. As shown in Fig. 2 and Supplemental Table 2, the higher the co-existence of multiple types of physical risk factors, the higher the odds of being obese and smoker (*p* for trend < 0.001). Workers who reported five or six types of physical risk factors had a 1.57 times higher odds of being obese (95%CI: 1.41–1.76) and a 1.52 times higher odds of being a smoker (95%CI: 1.40–1.65) compared to those without physical risk factors. No trend was found between the co-existence of multiple types of physical risk factors and physical inactivity during leisure time (*P* for trend = 0.82).

### Determinants of clustering of work-related risk factors and lifestyle factors

Workers with clustering of 4–6 physical risk factors, who were also obese and/or a smoker had a 7.94 (95%CI: 7.01–8.99) higher odds to be low educated than high educated (Table 2). Clustering of 2–3 psychosocial risk factors and being physically inactive or being a smoker also occurred most often among low educated workers (OR: 2.08, 95%CI: 1.93–2.25). No significant interactions were found between educational level and work-related risk factors, except for the association between physical risk factors and smoking, which was stronger among high educated workers than low educated workers (Supplemental Table 3, 4). The prevalence of multiple risk factors and unhealthy lifestyle factors was higher among low educated workers compared to high educated workers (Supplemental Tables 5, 6).

**Table 2:** Factors associated with the co-existence of multiple types of work-related risk factors and unhealthy lifestyle behaviors

	2–3 work-related psychosocial risk factors and being physically inactive and/or smoker vs. other	4–6 work-related physical risk factors and being obese and/or smoker vs. other
	*N* = 6549/46,014	*N* = 3421/49,142
Age (per 10 years)	**0.92 (0.90–0.95)**	**0.88 (0.85–0.91)**
Gender (female)	**1.20 (1.12–1.29)**	**0.83 (0.75–0.92)**
Education		
Low	**2.08 (1.93–2.25)**	**7.94 (7.01–8.99)**
Intermediate	**1.37 (1.29–1.46)**	**4.33 (3.87–4.85)**
High	Ref	Ref

Odds ratios for the associations between sociodemographic characteristics, and the co-existence of multiple types of work-related risk factors with unhealthy lifestyle behaviors. All factors were included in the same model and adjusted for irregular working hours and working hours per week

Boldface indicates statistical significance (*P* < 0.05)

Psychosocial risk factors and lifestyle clustered more often in women, while physical risk factors and lifestyle clustered more often among men (Table 2). Significant interaction between gender and work-related risk factors was observed in all associations, with most associations being stronger in men (Supplemental Tables 3, 4). The prevalence of psychosocial risk factors was slightly higher among women, and the prevalence of obesity was higher among men (Supplemental Tables 5, 6). Table 2 also shows that clustering of both psychosocial and physical work-related risk factors and lifestyle factors slightly decreased by age. No significant interaction with age was found, and there were only small differences in the prevalence of work-related risk factors and lifestyle factors between younger and older workers, except for obesity (Supplemental Table 5–6).

## Discussion

The findings of the present study partly confirm our hypothesis. The co-existence of multiple types of work-related psychosocial risk factors was significantly associated with a higher odds of being a smoker and being physically inactive but was not associated with obesity. Furthermore, the co-existence of multiple types of work-related physical risk factors was significantly associated with a higher odds of being a smoker and obese but not with physical inactivity during leisure time. It seems that there was no synergistic effect, meaning that the results did not show a significantly stronger association for the number of work-related risk factors than the sum of the strength of the associations between the separate risk factors and lifestyle factors. In addition, clustering of work-related risk factors and lifestyle was particularly common among low educated workers. Our results are largely in line with the results of a previous cross-sectional study among nursing home workers (Miranda et al. 2015). We extent those previous findings by showing that the associations differ by type of work-related risk factors, i.e., physical or psychosocial risk factors and we further searched for subgroups in which work-related risk factors and unhealthy lifestyle factors particularly cluster. The co-existence of multiple types of psychosocial and physical risk factors was associated with smoking, but only the co-existence of multiple types of psychosocial risk factors was associated with physical inactivity during leisure time and only the co-existence of multiple types of physical risk factors was associated with obesity. The absence of an association between psychosocial risk factors and obesity is in line with a meta-analysis, which described no or weak associations between single psychosocial risk factors and overweight (Sun et al. 2018). The absence of an association between physical risk factors and physical inactivity during leisure time may be partly attributed to misclassification because self-reported questionnaires were used (Sallis and Saelens 2000).

Several mechanisms might explain the observed associations between work-related risk factors and lifestyle. Clustering of multiple types of risk factors and smoking might be explained by findings showing that workers try to relieve mental work stress by smoking (Dobson et al. 2018; Ng and Jeffery 2003). Among workers in manual labor who have multiple physical risk factors, often a culture exists in which it is acceptable to smoke because relatively many coworkers smoke. As a consequence, people feel little pressure to quit smoking (Kouvonen et al. 2008). The lack of restrictions and the easy opportunities for workers in outdoor work to smoke may also contribute to clustering of multiple physical risk factors and smoking (Wardle and Steptoe 2003). A possible explanation for the association between physical risk factors and obesity might be that workers with physically demanding work eat more often comfort food and less healthy food and drinks due to increased tiredness caused by physically demanding work or the social culture at the workplace (Courtenay 2000; Engbers et al. 2006; Kolmet et al. 2006; Roos et al. 2007). A possible explanation for the association between psychosocial risk factors and physical inactivity during leisure time is that workers might find it difficult to exercise after a mentally demanding workday, mostly due to lack of time and energy (Payne et al. 2013; Roos et al. 2007).

Due to the cross-sectional design of this study, reversed causality may also explain our findings. People with an unhealthy lifestyle may more often end up in jobs with work-related risk factors than people with a healthy lifestyle (Mackenbach 2012). The materialist explanation suggests that circumstances such as financial resources, but also work-related risk factors, explain such health inequalities (Hoffmann et al. 2018; van der Beek and Kunst 2019). On the other hand, the behavioral explanation emphasizes that people who have poor health and, on average, an unhealthy lifestyle, more often have low-status positions compared to those with good health (Kröger et al. 2015; van der Beek and Kunst 2019). These explanations are underlined by several systematic reviews, which showed that both work-related risk factors and unhealthy lifestyle factors occur more often in people with a low than high socioeconomic position, and largely contribute to socioeconomic inequalities in health (Dieker et al. 2019; Hoven and Siegrist 2013; Moor et al. 2017). We also found that work-related risk factors clustered with obesity, smoking, and physical inactivity and that this clustering occurred, in particular, among low educated workers. Our analyses showed that clustering among low educated workers could be explained by the higher prevalence of work-related risk factors among these workers and not because the strength of the associations between work-related risk factors and lifestyle differed between low and high educated workers.

The clustering of work-related risk factors and unhealthy lifestyle factors among younger aged workers was unexpected and could not be fully explained in our analyses. We found no interaction by age in the associations, but differences by age could be partly explained by the higher prevalence of work-related physical risk factors among younger workers compared to older workers. For gender, we also found relatively small differences in clustering of work-related risk factors and lifestyle between male and female workers. Our analyses showed stronger associations between physical risk factors and smoking among male workers compared to female workers, which may explain partly why clustering of physical risk factors occurred more often among male workers. The findings that the association between psychosocial risk factors and lifestyle was also stronger among male workers indicated that male workers might cope less well with work-related risk factors, which may need particular attention in prevention programs. The prevalences of work-related risk factors were slightly higher among female workers, which may partly explain why psychosocial risk factors and lifestyle cluster more often among female workers. More research is needed to further explain the observed differences by age and gender.

The clustering of work-related risk factors and unhealthy lifestyle factors among workers with a low education level highlights the importance to target prevention with an integral approach to these groups. As work-related psychosocial and physical risk factors and unhealthy lifestyle factors co-occur in the same group of workers it seems insufficient to focus preventive efforts on a single factor. Studies also show that workplace health promotion programs which address all factors simultaneously are more successful in improving the health of workers than programs focusing on single risk factors (Feltner et al. 2016; Goldgruber and Ahrens 2010; Schröer et al. 2013). With regard to low educated workers, workplace health promotion programs should be tailored and developed to the needs of these type of workers, since most available programs do not reach low educated workers and are less effective for low than high educated workers (Cairns et al. 2014; Groeneveld et al. 2010; Puhkala et al. 2015). This indicates that more insight in the needs and possibilities of low educated workers is necessary, for example with the use of participatory approaches. Participatory approaches actively engage the target population in the development and implementation of a workplace health promotion program, to be able to design a program that fits with the needs of the target population (Goldgruber and Ahrens 2010; Lingard and Turner 2015). Accordingly, further research is needed to develop and evaluate effective programs for workers with a low educational level, by using participatory methods that address simultaneously physical and psychosocial risk factors, as well as unhealthy lifestyle factors.

A strength of the present study is the extensive measurement of several work-related physical and psychosocial risk factors and the large sample size, leading to large statistical power. This enabled us to investigate differences by psychosocial and physical risk factors in their association with obesity and unhealthy lifestyle behaviors. The present study has also several limitations. First, due to the cross-sectional study design it was not possible to determine the causal direction of associations between work-related risk factors and outcomes. However, the aim of the present study was not to investigate the causal relation, but to investigate the occurrence of risk factors and the outcomes at the same time (i.e., clustering) and to identify groups of workers in whom this clustering occurred most often. The second limitation is that participants in the current study were classified as being obese, smoker and physically inactive based on self-reported data. Although self-reported smoking status and body weight have a moderately good reliability (Dekkers et al. 2008; Huerta et al. 2005), participants may not have reported that they smoke, overestimated their physical activity level, and obese participants may have underestimated their body weight (Adams et al. 2005; Dyrstad et al. 2014; Gorber et al. 2007). Consequently, it is likely that the associations between the co-existence of multiple types of risk factors and unhealthy lifestyle factors were underestimated.

The co-existence of multiple types of work-related psychosocial risk factors was associated with a higher odds to be a smoker and to be physically inactive. Workers with co-existing physical risk factors were more likely to smoke and to be obese. Clustering of multiple work-related risk factors and unhealthy lifestyle factors occurred most often in workers with a low educational level. This study highlights the need for an integral approach of prevention that takes into account the co-existence of work-related psychosocial and physical risk factors as well as lifestyle factors in low educated workers.

## Supplementary Information

Below is the link to the electronic supplementary material.

Supplementary file1 (DOCX 21 KB)

## Data Availability

Due to ethical restrictions related to participant consent and sensitivity of the company data, all relevant data are under the conditions of Bewegen Werkt available upon request to the responsible advisor Marike van Kalken (Marike.vanKalken@bewegenwerkt.nl).

## References

[CR1] Adams SA, Matthews CE, Ebbeling CB, Moore CG, Cunningham JE, Fulton J, Hebert JR (2005). The effect of social desirability and social approval on self-reports of physical activity. Am J Epidemiol.

[CR2] Alavinia SM, Van Den Berg TI, Van Duivenbooden C, Elders LA, Burdorf A (2009). Impact of work-related factors, lifestyle, and work ability on sickness absence among Dutch construction workers. Scand J Work Environ Health.

[CR3] Bambra C, Gibson M, Sowden A, Wright K, Whitehead M, Petticrew M (2009). Working for health? Evidence from systematic reviews on the effects on health and health inequalities of organisational changes to the psychosocial work environment. Prev Med.

[CR4] Brunner EJ, Chandola T, Marmot MG (2007). Prospective effect of job strain on general and central obesity in the Whitehall II Study. Am J Epidemiol.

[CR5] Buckwalter JA (1995). Aging and degeneration of the human intervertebral disc. Spine.

[CR6] Cairns J-M, Bambra C, Hillier-Brown FC, Moore HJ, Summerbell CD (2014). Weighing up the evidence: a systematic review of the effectiveness of workplace interventions to tackle socio-economic inequalities in obesity. J Pub Health.

[CR7] Cooper R (2011). Age and gender differences in physical capability levels from mid-life onwards: the harmonisation and meta-analysis of data from eight UK cohort studies. PLoS ONE.

[CR8] Courtenay WH (2000). Engendering health: A social constructionist examination of men’s health beliefs and behaviors. Psychol Men Masc.

[CR9] Danaei G, Ding EL, Mozaffarian D, Taylor B, Rehm J, Murray CJ, Ezzati M (2009). The preventable causes of death in the United States: comparative risk assessment of dietary, lifestyle, and metabolic risk factors. PLoS Med.

[CR10] Danaei G (2014). Cardiovascular disease, chronic kidney disease, and diabetes mortality burden of cardiometabolic risk factors from 1980 to 2010: a comparative risk assessment. Lancet Diabetes Endocrinol.

[CR11] Dawson KM, O'Brien KE, Beehr TA (2016). The role of hindrance stressors in the job demand–control–support model of occupational stress: a proposed theory revision. J Organ Behav.

[CR12] De Jonge J, Kompier MA (1997). A critical examination of the demand-control-support model from a work psychological perspective. Internat J Stress Manage.

[CR13] Dekkers JC, van Wier MF, Hendriksen IJ, Twisk JW, van Mechelen W (2008). Accuracy of self-reported body weight, height and waist circumference in a Dutch overweight working population. BMC Med Res Method.

[CR14] Dieker AC, Proper KI, Burdorf A, Ket JC, van der Beek AJ, Hulsegge G (2019). The contribution of work and lifestyle factors to socioeconomic inequalities in self-rated health a systematic review. Scand J Work Environ Health.

[CR15] Dobson KG, Gilbert-Ouimet M, Mustard CA, Smith PM (2018). Association between dimensions of the psychosocial and physical work environment and latent smoking trajectories: a 16-year cohort study of the Canadian workforce. Occup Environ Med.

[CR16] Dumith SC, Hallal PC, Reis RS, Kohl HW (2011). Worldwide prevalence of physical inactivity and its association with human development index in 76 countries. Prev Med.

[CR17] Dyrstad SM, Hansen BH, Holme IM, Anderssen SA (2014). Comparison of self-reported versus accelerometer-measured physical activity. Med Sci Sports Exerc.

[CR18] Engbers LH, van Poppel MN, Paw MCA, van Mechelen W (2006). The effects of a controlled worksite environmental intervention on determinants of dietary behavior and self-reported fruit, vegetable and fat intake. BMC Public Health.

[CR19] Feltner C, Peterson K, Weber RP, Cluff L, Coker-Schwimmer E, Viswanathan M, Lohr KN (2016). The effectiveness of Total Worker Health interventions: a systematic review for a National Institutes of Health pathways to prevention workshop. Ann Intern Med.

[CR20] Golden L. Irregular work scheduling and its consequences. Economic Policy Institute Briefing Paper. 2015.

[CR21] Goldgruber J, Ahrens D (2010). Effectiveness of workplace health promotion and primary prevention interventions: a review. J Pub Health.

[CR22] Gorber SC, Tremblay M, Moher D, Gorber B (2007). A comparison of direct vs. self-report measures for assessing height, weight and body mass index: a systematic review. Obes Rev.

[CR23] Groeneveld IF, Proper KI, Van der Beek AJ, Van Mechelen W (2010). Sustained body weight reduction by an individual-based lifestyle intervention for workers in the construction industry at risk for cardiovascular disease: results of a randomized controlled trial. Prev Med.

[CR24] Hansson RO, Robson SM, Limas MJ (2001). Stress and coping among older workers. Work.

[CR25] Häusser JA, Mojzisch A, Niesel M, Schulz-Hardt S (2010). Ten years on: a review of recent research on the Job Demand-Control (-Support) model and psychological well-being. Work Stress.

[CR26] Heikkilä K (2012). Job strain and tobacco smoking: an individual-participant data meta-analysis of 166 130 adults in 15 European studies. PLoS ONE.

[CR27] Heikkilä K (2013). Job strain and health-related lifestyle: findings from an individual-participant meta-analysis of 118 000 working adults. Am J Pub Health.

[CR28] Hoffmann R, Kröger H, Pakpahan E (2018). Pathways between socioeconomic status and health: Does health selection or social causation dominate in Europe?. Adv Life Course Res.

[CR29] Holtermann A (2010). Worksite interventions for preventing physical deterioration among employees in job-groups with high physical work demands: background, design and conceptual model of FINALE. BMC Public Health.

[CR30] Hooftman W, Jong Td, Vos F. Monitoring NPDI 2014: overzicht en verdiepende analyses. TNO. 2014

[CR31] Hoven H, Siegrist J (2013). Work characteristics, socioeconomic position and health: a systematic review of mediation and moderation effects in prospective studies. Occup Environ Med.

[CR32] Huerta M, Chodick G, Balicer RD, Davidovitch N, Grotto I (2005). Reliability of self-reported smoking history and age at initial tobacco use. Prev Med.

[CR33] Jaaskelainen A (2015). Psychosocial factors at work and obesity among young finnish adults: a cohort study. J Occup Environ Med.

[CR34] Kenny GP, Yardley JE, Martineau L, Jay O (2008). Physical work capacity in older adults: implications for the aging worker. Am J Ind Med.

[CR35] Kolmet M, Marino R, Plummer D (2006). Anglo-Australian male blue-collar workers discuss gender and health issues International. J Men Health.

[CR36] Kouvonen A (2008). Work-place social capital and smoking cessation: the finnish public sector study. Addiction.

[CR37] Kouvonen A (2013). Chronic workplace stress and insufficient physical activity: a cohort study. Occup Environ Med.

[CR38] Kröger H, Pakpahan E, Hoffmann R (2015). What causes health inequality? A systematic review on the relative importance of social causation and health selection. Eur J Pub Health.

[CR39] Lingard H, Turner M (2015). Improving the health of male, blue collar construction workers: a social ecological perspective. Construct Manage Econ.

[CR40] Mackenbach JP (2012). The persistence of health inequalities in modern Welfare states: the explanation of a paradox. Soc Sci Med.

[CR41] Mäkinen T, Kestilä L, Borodulin K, Martelin T, Rahkonen O, Leino-Arjas P, Prättälä R (2010). Occupational class differences in leisure-time physical inactivity-contribution of past and current physical workload and other working conditions. Scand J work Environ Health.

[CR42] Marques A, Peralta M, Naia A, Loureiro N, de Matos MG (2017). Prevalence of adult overweight and obesity in 20 European countries, 2014. Eur J Public Health.

[CR43] Miranda H, Gore RJ, Boyer J, Nobrega S, Punnett L (2015). Health behaviors and overweight in nursing home employees: contribution of workplace stressors and implications for worksite health promotion. Scient World J.

[CR44] Moor I, Spallek J, Richter M (2017). Explaining socioeconomic inequalities in self-rated health: a systematic review of the relative contribution of material, psychosocial and behavioural factors. J Epidemiol Commun Health.

[CR45] Morassaei S, Smith PM (2011). Examining the relationship between psychosocial working conditions, physical work demands, and leisure time physical activity in Canada. J Occup Environ Med.

[CR46] Ng DM, Jeffery RW (2003). Relationships between perceived stress and health behaviors in a sample of working adults. J Health Psychol.

[CR47] Nobrega S (2016). Obesity/overweight and the role of working conditions: a qualitative, participatory investigation. Health Promo Pract.

[CR48] Payne N, Jones F, Harris PR (2013). Employees’ perceptions of the impact of work on health behaviours. J Health Psychol.

[CR49] Puhkala J (2015). Lifestyle counseling to reduce body weight and cardiometabolic risk factors among truck and bus drivers–a randomized controlled trial. Scand J Work Environ Health.

[CR50] Radi S, Ostry A, LaMontagne AD (2007). Job stress and other working conditions: Relationships with smoking behaviors in a representative sample of working Australians. Am J Ind Med.

[CR51] Reitsma MB (2017). Smoking prevalence and attributable disease burden in 195 countries and territories, 1990–2015: a systematic analysis from the Global Burden of Disease Study 2015. Lancet.

[CR52] Robroek SJ, van Lenthe FJ, Burdorf A (2013). The role of lifestyle, health, and work in educational inequalities in sick leave and productivity loss at work. Int Arch Occup Environ Health.

[CR53] Roh L, Braun J, Chiolero A, Bopp M, Rohrmann S, Faeh D (2014). Mortality risk associated with underweight: a census-linked cohort of 31,578 individuals with up to 32 years of follow-up. BMC Public Health.

[CR54] Roos E, Sarlio-Lähteenkorva S, Lallukka T, Lahelma E (2007). Associations of work–family conflicts with food habits and physical activity. Public Health Nutr.

[CR55] Sallis JF, Saelens BE (2000). Assessment of physical activity by self-report: status, limitations, and future directions. Res Quart Exercise Sport.

[CR56] Schneider S, Becker S (2005). Prevalence of physical activity among the working population and correlation with work-related factors: results from the first German National Health Survey. J Occup Health.

[CR57] Schröer S, Haupt J, Pieper C (2013). Evidence-based lifestyle interventions in the workplace—an overview. Occup Med.

[CR58] Sjöström M, Oja P, Hagströmer M, Smith B, Bauman A (2006). Health-enhancing physical activity across European Union countries: the Eurobarometer study. J Public Health.

[CR59] Strazdins L, Bammer G (2004). Women, work and musculoskeletal health. Soc Sci Med.

[CR60] Sun M (2018). Meta-analysis on shift work and risks of specific obesity types. Obes Rev.

[CR61] Törner M, Blide G, Eriksson H, Kadefors R, Karlsson R, Petersen I (1988). Musculo-skeletal symptoms as related to working conditions among Swedish professional fisherman. Appl Ergon.

[CR62] van der Beek AJ, Kunst AE (2019). How can we break the vicious circle between poor health and exit from paid employment?. Scand J Work Environ Health.

[CR63] van der Beek AJ (2017). A research framework for the development and implementation of interventions preventing work-related musculoskeletal disorders. Scand J Work Environ Health.

[CR64] Wardle J, Steptoe A (2003). Socioeconomic differences in attitudes and beliefs about healthy lifestyles. J Epidemiol Commun Health.

[CR65] WHO (2000). Obesity: preventing and managing the global epidemic: Report of a WHO consultation.

[CR66] Winkler MR, Mason S, Laska MN, Christoph MJ, Neumark-Sztainer D (2018). Does non-standard work mean non-standard health? Exploring links between non-standard work schedules, health behavior, and well-being. SSM Popul Health.

