# Supplementary material for: Work-related physical and psychosocial risk factors cluster with obesity, smoking and physical inactivity
Source: Int Arch Occup Environ Health. 2021 Jan 6;94(4):741–50. doi: 10.1007/s00420-020-01627-1 (PMC8068657; doi:10.1007/s00420-020-01627-1)
Supplement: Supplementary file 1 — Supplementary file1 (DOCX 21 KB) [file 420_2020_1627_MOESM1_ESM.docx]

|  | **Obesity** |  | **Smoking** | **Physical activity** |
| --- | --- | --- | --- | --- |
| 0 work factors | ref |  | ref | ref |
| 1 | **1.00 (0.94-1.07)** |  | **1.00 (0.95-1.05)** | **1.20 (1.15-1.25)** |
| 2 | **1.04 (0.96-1.12)** |  | **1.08 (1.02-1.15)** | **1.32 (1.25-1.39)** |
| 3 | **1.09 (0.95-1.25)** |  | **1.23 (1.11-1.36)** | **1.55 (1.42-1.70)** |

**Supplemental Table 1.** Associations between the number of work-related psychosocial risk factors and obesity, smoking and physical inactivity during leisure time presented

Analyses were adjusted for gender, age, working hours per week and irregular working hours.
Note: Sum of work-related psychosocial risk factors: low decision authority, low skill discretion, and high job demands
*0* psychosocial risk factors = reference category
* *p < 0.05*

**Supplemental Table 2.** Associations between the number of work-related physical risk factors and obesity, smoking and physical inactivity during leisure time presented

|  | **Obesity** | **Smoking** | **Physical activity** |
| --- | --- | --- | --- |
| 0 work factors | ref | ref | ref |
| 1 | **1.22 (1.11-1.34)** | **0.98 (0.91-1.05)** | **1.09 (1.03-1.15)** |
| 2 | **1.30 (1.20-1.42)** | **0.98 (0.91-1.04)** | **1.12 (1.06-1.18)** |
| 3 | **1.35 (1.22-1.49)** | **1.27 (1.82-1.37)** | **0.97 (0.91-1.04)** |
| 4 | **1.30 (1.16-1.45)** | **1.38 (1.27-1.18)** | **1.05 (0.98-1.13)** |
| 5-6 | **1.57 (1.41-1.76)** | **1.52 (1.40-1.65)** | **1.01 (0.93-1.09)** |

Analyses were adjusted for gender, age, working hours per week and irregular working hours.
Note: Sum of work-related physical risk factors: lifting or moving heavy loads, awkward working postures, applying force with arms or hands, frequently bending and/or twisting the upper body, frequently working in the same position, and repetitive movements with arms and/or hands.
*0* physical risk factors = reference category
* *p < 0.0*

**Supplemental Table 3.** The associations between work-related psychosocial risk factors, smoking and physical inactivity stratified by gender

|  | 0 risk factors | 1 risk factor | 2 risk factors | 3 risk factors | |
| --- | --- | --- | --- | --- | --- |
|  | **Smoking OR (95%)** | | | |  |
| Male | ref | 0.95  (0.88-1.03) | 1.11  (1.01-1.22) | 1.44  (1.20-1.72) | |
| Female | ref | 1.02  (0.95-1.09) | 1.05  (0.97-1.14) | 1.13  (1.00-1.28) | |
|  | **Physical inactivity OR (95%)** | | | | |
| Male | ref | 1.26  (1.18-1.34) | 1.38  (1.27-1.50) | 1.62  (1.38-1.90) | |
| Female | ref | 1.15  (1.09-1.22) | 1.28  (1.19-1.36) | 1.51 (1.36-1.69) | |

Analyses were adjusted for age, working hours per week and irregular working hours.
* Significant interactions between gender and psychosocial risk factors in relation with smoking and physical inactivity was found.

**Supplemental Table 4.** The associations between work-related physical risk factors, smoking and obesity stratified by gender and educational level

|  | 0 risk factors | 1 risk factor | 2 risk factors | 3 risk factors | 4 risk factors | 5-6 risk factors |
| --- | --- | --- | --- | --- | --- | --- |
| Smoking OR (95%) | | | | | | |
| High/middle educated | ref | 0.94  (0.87-1.02) | 0.95 (0.89-1.03) | 1.21 (1.11-1.31) | 1.30  (1.18-1.42) | 1.41  (1.28-1.56) |
| Low educated | ref | 0.99  (0.82-1.19) | 0.90 (0.75-1.06) | 1.02 (0.85-1.21) | 1.09  (0.90-1.31) | 1.09 (0.91-1.30) |
|  |  | | | | | |
| Male | ref | 0.98 (0.88-1.10) | 0.94 (0.85-1.05) | 1.40 (1.25-1.58) | 1.75 (1.53-2.00) | 1.91 (1.67-2.17) |
| Female | ref | 0.97 (0.88-1.07) | 0.98 (0.90-1.07) | 1.20 (1.09-1.31) | 1.23 (1.11-1.36) | 1.32 (1.19-1.47) |
| Obesity  OR (95%) | | | | | | |
| Male | ref | 1.08 (0.99-1.17) | 1.13 (1.05-1.23) | 1.14 (1.03-1.27) | 0.99 (0.88-1.12) | 1.35 (1.20-1.52) |
| Female | ref | 1.08  (1.01-1.17) | 1.24 (1.16-1.33) | 1.22 (1.13-1.32) | 1.30 (1.19-1.41) | 1.36 (1.24-1.48) |

Analyses were adjusted for age, working hours per week and irregular working hours. Analyses for educational level were also adjusted for gender.
* Significant interactions of educational level and gender with physical risk factors was found in relation to smoking. In the association with obesity, significant interactions were observed for gender.

| **Psychosocial risk factors** | | | |  | **Physical risk factors** | | | | | |
| --- | --- | --- | --- | --- | --- | --- | --- | --- | --- | --- |
|  | 0 rf | 1 rf | 2 rf | 3 rf | 0 rf | 1 rf | 2 rf | 3 rf | 4 rf | 5-6 rf |
| **High/Middle educated** | 33.6% (15012) | 41.7% (18643) | 20.3% (9091) | 4.4% (1954) | 22.8% (10205) | 19% (8487) | 27.8% (12409) | 13.8% (6149) | 9.2% (4133) | 7.4% (3317) |
| **Low educated** | 28.4% (2233) | 38.2% (3006) | 26.9% (2119) | 6.4% (505) | 13.3% (1045) | 15.1% (1191) | 22.2% (1744) | 19% (1495) | 14.2% (1113) | 16.2%  (1275) |
|  |  |  |  |  |  |  |  |  |  |  |
| **Male** | 36.6% (7375) | 41.6% (8388) | 18.5% (3728) | 3.3% (662) | 23% (4637) | 21.9% (4421) | 28.2% (5683) | 11.6% (2332) | 7.4% (1493) | 7.9% (1587) |
| **Female** | 30.5% (9870) | 40.9% (13261) | 23.1% (7482) | 5.5% (1797) | 20.4% (6613) | 16.2% (5257) | 26.1% (8470) | 16.4% (5312) | 11.6% (3753) | 9.3% (3005) |
|  |  |  |  |  |  |  |  |  |  |  |
| **Age ≤ 45 years** | 31.7% (8641) | 42.3% (11534) | 21.4% (5379) | 4.5% (1226) | 19.7% (5369) | 18% (4916) | 27.1% (7388) | 15.4% (4188) | 10.4% (2843) | 9.3% (2535) |
| **Age >45 years** | 34% (8604) | 39.9% (10115) | 21.2% (5379) | 4.8% (1226) | 23.2% (5881) | 18.8% (9678) | 26.7% (14153) | 13.6% (7644) | 9.5% (2403) | 8.1% (2057) |

**Supplemental Table 5.** Prevalence of work-related psychosocial and physical risk factors stratified by educational level, gender and age

|  | **Smoking** | **Obesity** | **Physical inactivity** |
| --- | --- | --- | --- |
| **High/Middle educated** | 18% (8060) | 44.2% (19757) | 33% (14735) |
| **Low educated** | 30.2% (2371) | 56.4% (4435) | 33.8% (2661) |
|  | | | |
| **Male** | 21% (4240) | 56.5% (11385) | 38.1% (7681) |
| **Female** | 19.1% (6191) | 39.5% (12807) | 30% (9715) |
|  | | | |
| **Age ≤45 years** | 20.3% (5516) | 40.3% (10974) | 35.1% (9557) |
| **Age > 45 years** | 19.4% (4915) | 52.2% (13218) | 31% (7839) |
|  | | | |

**Supplemental Table 6.** Prevalence of smoking, obesity and physical inactivity stratified by educational level, gender, and age
